# A pilot randomised controlled trial of physical activity facilitation for older adults: feasibility study findings

**DOI:** 10.1186/s40814-019-0414-9

**Published:** 2019-03-08

**Authors:** Gemma S. Morgan, Anne M. Haase, Rona M. Campbell, Yoav Ben-Shlomo

**Affiliations:** 1 Population Health Sciences, Bristol Medical School, University of Bristol, Bristol, UK; 2 School of Policy Studies, University of Bristol, Bristol, UK

**Keywords:** Physical activity, Ageing, Disability, Randomised controlled trial, Complex intervention, Self-determination theory, Physical performance

## Abstract

**Background:**

More people are living longer lives leading to a growth in the population of older adults, many of whom have comorbidities and low levels of physical function. Physical activity in later life can prevent or delay age-related disability. Identifying a cost-effective means of increasing physical activity in older adults therefore remains an important public health priority.

Physical Activity Facilitation (PAF) is an intervention shown to increase physical activity in adults with depression. The PAF model was modified for a population of older adults at risk of disability. This study aimed to assess the feasibility of undertaking a definitive RCT of the PAF intervention in the target population.

**Methods:**

A pilot randomised controlled trial (RCT) was delivered through primary care. Patients at risk of disability and who were not meeting recommended levels of physical activity were recruited through postal invitation and direct approach in the practice waiting room. Those meeting eligibility criteria were enrolled and randomised at a 2:1 ratio to the PAF intervention and control. Behaviour change techniques were used by facilitators with participants over the telephone and face-to-face for 6 months. Outcome measures including physical function, physical activity, depression, social support, and quality of life were collected at baseline and at 6 months.

**Results:**

A high proportion of patients responded to the initial invitation (68%), yet many were ineligible due to high levels of self-reported physical activity and baseline physical function. Fifty-one participants were recruited to the trial, with an average age of 74 years (range 65–89), and there were high rates of adherence and retention to the study (94% follow-up at 6 months). The majority of outcome data collected from participants was complete; however, the validated scale used to measure self-reported physical activity was associated with high levels of missing data.

**Conclusions:**

The findings of this pilot RCT suggest that it is feasible to deliver a definitive RCT of the PAF intervention in this population. Further work is required to improve the efficiency of recruitment and to minimise missing data from self-reported physical activity measures.

**Trial registration:**

Current controlled trials ISRCTN80470273. Registered 25 October 2013.

**Electronic supplementary material:**

The online version of this article (10.1186/s40814-019-0414-9) contains supplementary material, which is available to authorized users.

## Background

Recent advances in medical science and public health have reduced overall premature mortality, resulting in a larger population of older adults with increasing prevalence of comorbidities. In the UK in 2016, adults aged 65 years and over comprised 18% of the population and it is projected that this will increase to 25% over the next 30 years [1]. It is therefore important that we identify ways of enabling older people to function well and live independently as they age. Findings from the LIFE study [2] support the hypothesis that physical activity in later life can prevent or delay age-related disability. However, we know that only around half of 65–74 year olds in the UK report doing sufficient activity to meet recommended targets [3, 4] and physical activity levels in those aged 75 years and older are even lower [5]. Identifying a cost-effective and sustainable means of increasing physical activity in older adults therefore remains an important public health priority.

An existing intervention, Physical Activity Facilitation [6], has been used successfully to increase physical activity in adults with depression [7]. The PAF intervention was modified and developed for application to a population of older adults at risk of disability. The intervention uses facilitators to deliver behaviour change techniques with motivational interviewing strategies [8]. In summary, the PAF facilitator acts as the main agent of change and aims to address the core psychological needs of participants through face-to-face and telephone sessions. It is hypothesised that the PAF intervention will be effective in increasing physical activity in older adults at risk of disability, and that the increased physical activity will lead to a reduction in disability and/or improved physical performance.

### Aim and objectives

The aim of this study was to assess the feasibility of undertaking a definitive and full-scale RCT of the PAF intervention in the target population. There were four objectives linked to the pilot RCT:

i) Test the effectiveness of methods of recruitment and enrolment
ii) Assess retention and adherence to the study and/or intervention
iii) Evaluate the methods of data collection and analysis for a definitive trial
iv) Provide estimates of the variability of key outcomes to enable estimation of the sample size and resources required for a future definitive trial

A series of criteria, indicating what needed to be achieved in the pilot and feasibility for there to be progression to a definitive trial, were prespecified (see Table 1). In addition, a mixed methods process evaluation was nested within the pilot RCT to assess implementation and acceptability including adverse events, mechanism of impact, and context. Detailed methods and findings from the process evaluation will be published separately.

**Table 1:** Pre-specified feasibility criteria

Criteria for proceeding to definitive trial	Assessment	Linked objective
An acceptable proportion of individuals respond to recruitment invitations.	Recruitment records show an initial response rate of > 10% to postal invitation and/or > 25% to primary care practitioner invitation or waiting room recruitment. The three methods will be compared.	(1) Test the effectiveness of methods of recruitment and enrolment
An acceptable proportion of individuals responding to recruitment invitations are eligible to participate.	Screening records show a screen-failure rate of < 80%.	(1) Test the effectiveness of methods of recruitment and enrolment
Attrition from the pilot trial is low.	Trial records show that the proportion of enrolled participants “lost to follow-up” at 6 months is < 20%, excluding deaths and long-term care or hospitalisation.	(2) Assess retention and adherence to the study and/or intervention
PAF intervention attracts high rates of participation from eligible adults.	Trial records indicate that adherence to the intervention is high; ≥ 65% of intervention participants participate in at least one face-to-face and five telephone sessions.	(2) Assess retention and adherence to the study and/or intervention
PAF can engage individuals from a range of socio-economic localities.	Participants are recruited from primary care practices in wards with high deprivation scores and low deprivation scores.	(1) Test the effectiveness of methods of recruitment and enrolment
PAF delivery costs can be recorded in a way that enables cost-effectiveness analysis.	Systems developed in the exploratory trial can be used to monitor the costs of a definitive RCT.	(3) Evaluate the methods of data collection and analysis for a definitive trial
Methods for measuring primary and secondary outcomes and mediator variables are feasible and acceptable.	Process evaluation findings and completed questionnaires suggest that self-report and objective measures were comprehensible and acceptable to > 80% of participants.	(3) Evaluate the methods of data collection and analysis for a definitive trial
The sample size required for an adequately powered trial is achievable.	Measurement variability of the primary outcome, recruitment rates, and expected attrition are consistent with a sample size that can be achieved within a reasonable time.	(4) Provide estimates of the variability of key outcomes to enable estimation of the sample size and resources required for a future definitive trial

## Methods

The study protocol has been published previously [9]; methods for the pilot trial are summarised below.

### Recruitment and randomisation

The initial recruitment target was 60 adults aged 65 years and over from six primary care practices based in areas of differing social deprivation across the South West area. This sample size was a pragmatic decision to ensure that adequate participants were available to estimate the key parameters [10]; recruitment closed at 51 participants. Recruitment ran for 9 months from April 2014 to January 2015 using a combination of postal invitation and opportunistic recruitment of patients from the practice waiting room.

### Recruitment by postal invitation

Practice staff identified an initial list of patients, randomly selecting a sample of those aged 65 and older and using “read codes” (codes used in electronic records to categorise patients and consultations) to exclude those with medical conditions listed in the exclusion criteria (see Table 2 for full eligibility criteria). General practitioners (GPs) reviewed the lists, and the final sample of patients was sent recruitment materials in the post. A single reminder invitation was sent to non-responders. Subjects expressing an interest in the study were followed up with a brief telephone screening call, and those reporting eligibility against broad criteria were invited to a screening clinic for full eligibility assessment (see Additional file 1: Figure S1 for details of questions asked during telephone screening).

**Table 2:** Participant eligibility criteria

Inclusion criteria	•	Aged 65 years or older
	•	Living in the community; this includes those living in sheltered accommodation
	•	Not meeting recommended levels of physical activity, defined as less than 150 min of moderate, or 75 min of vigorous, physical activity per week
	•	Not disabled at baseline, defined using a walking speed of at least 0.8 m/s along a 4-m walk track.
	•	At risk of subsequent disability, defined as a score of less than 10 out of 12 on the SPPB
Exclusion criteria	•	Unable to participate in the intervention or study due to speech, language, or sensory problems
	•	Resident in a nursing home
	•	Intention to move out of the study area within 6 m of the screening clinic visit or to be away for more than 8 consecutive weeks during this period
	•	Concurrent participation in an “exercise-on-prescription” or a physical rehabilitation programme or study
	•	A documented or patient-reported medical condition including but not limited to severe, uncontrolled arthritis; severe lung disease requiring regular use of corticosteroids or supplemental oxygen; serious cardiovascular disease; history of cardiac arrest; neuromuscular or musculoskeletal conditions exacerbated by exercise; moderate or severe cognitive impairment or dementia; severe uncontrolled psychiatric illness; and multiple (≥ 2) falls in previous 3 months
	•	Investigator concern about an individual’s safety or ability to adhere to the intervention if enrolled in the trial

*m/s* metres per second, *SPPB* Short Physical Performance Battery

### Recruitment in the waiting room

A large poster and leaflets in the practice informed patients that a researcher was present and recruiting for a study. Patients who appeared to broadly fit the eligibility criteria, as mentioned above, were approached, and those agreeing to speak with the researcher were asked the same broad eligibility questions asked during telephone screening (see Additional file 1: Figure S1). Individuals meeting the eligibility criteria were provided with a brief explanation of the study and an information leaflet. Those providing contact details at the time were called a few days later to arrange a screening clinic appointment for full eligibility assessment, and those expressing an interest but not happy to provide contact details were given a reply slip to return to the study team.

### Screening

As the PAF intervention has been designed as a preventive intervention, the target population were those who were non-disabled at baseline, but who were at risk of developing disability. As detailed in Table 2, to be eligible, participants were required (1) to be walking independently at baseline and (2) to have a level of functional performance that indicated an increased risk of disability. At the screening clinic, potential participants were screened for these two criteria.

#### Walking independently at baseline

There is evidence that the ability to complete a 4-m walking test (4MWT) at usual pace at a speed of 0.8 m/s or greater is highly predictive [11] of successfully completing the commonly used 400-m walk test (400MWT) [12] which is valid [11] and reliable [2]. Due to space limitations, the 4MWT was used as an alternative to the 400MWT and 0.8 m/s was used as the minimal threshold to be deemed walking independently at baseline. It was observed that a significant proportion of potential participants spent time “warming up” from a stationary position. As it was not specified whether the 4-m measurement should be taken from a standing start or from a mid-stride position, we included a short (1–2 m) “run-up” before the stopwatch was started. Times were recorded to the nearest hundredth of a second.

#### Assessing risk of mobility disability in the future

The Short Physical Performance Battery (SPPB) assesses elements of lower limb function and is a composite measure of walking speed (along a 4-m track), chair rises, and balance. The summary scores range from 1 (indicating lowest performance) to 12 (highest performance). The SPPB has been shown to be reliable [13], associated with disability-related outcomes including nursing home admission [14], and predictive of future new-onset disability, both self-reported and objective [15]. A systematic review of the predictive value of the SPPB concluded that individuals scoring less than 10 out of 12 have up to a 5-fold increased risk of new-onset disability [16]; this was used as the threshold for inclusion in the pilot RCT.

### Confirming exclusion criteria

Participants were asked to confirm the absence of each exclusion criterion listed in Table 2 before enrolment; these were subsequently corroborated with each participant’s GP. Eligible participants who provided consent to enrol in the study completed further baseline assessments (physical assessments and paper-based surveys) and were randomly allocated, using a computer-based randomisation system, to either the PAF intervention or usual care at a 2:1 ratio. The 2:1 ratio was chosen to ensure adequate numbers of intervention participants remained in the study in order to ensure rich data collection in the process evaluation. This approach is an accepted method of ensuring adequate retention in the intervention arm [17]. To ensure similar distribution of participant characteristics between each arm, a minimisation algorithm was used, minimising on age, gender, and GP practice.

### Intervention and control arms

The PAF intervention is based upon self-determination theory [18], which asserts that for an individual to modify their behaviour, three core psychological needs must be met: the need for autonomy (having control and choice over activity), for competence (feeling capable about doing something), and for relatedness (feeling connected to and supported by others). Evidence-based behaviour change techniques [19] derived from control theory such as action planning, goal setting, self-monitoring of behaviour, feedback, and goal reviewing were used during sessions, and where possible, the PAF facilitators aimed to focus on “lifestyle physical activity”, i.e. that which fits into an individual’s day-to-day life and daily routine. Each participant randomised to the intervention was offered an initial face-to-face PAF session, up to two further face-to-face sessions, and up to nine telephone support sessions over the 6-month intervention period. Sessions were not at fixed intervals but were tailored to suit the individual participant and their progress. Worksheets designed to assist with behaviour change techniques were available for facilitators and participants to use. PAF facilitators were recruited locally; previous experience working with older adults or in health or social care was desirable but not essential. A 3-day training course and comprehensive training manual were provided at the start of the study, and regular supervision was provided throughout the intervention period.

Participants randomised to the control arm were provided with a booklet which contained advice on healthy ageing and which was publicly available, published by a national voluntary organisation.

### Study outcomes

The primary quantitative outcomes were measures of feasibility to inform the design of a future full-scale trial, i.e. effectiveness of recruitment strategies, measures of retention and adherence, and the feasibility of collecting outcome measures. It is not appropriate to conduct hypothesis testing around effectiveness in an underpowered pilot study. However, the methods used for evaluating the intervention in a definitive trial were replicated in this pilot study, as a means of testing the feasibility of data collection and analysis and to identify the standard deviation of key variables, which may be used as primary outcomes in powering a future definitive trial. Variables most likely to be considered as a primary outcome in a definitive trial include mean walking speed; SPPB score; and daily minutes of light, moderate-to-vigorous, and sedentary activity. Walking speed and performance on the SPPB were measured as part of the screening clinic and were repeated by a trained nurse or healthcare assistant, who was blinded to the treatment allocation, at a follow-up clinic held 6 months after enrolment. Measures of objective physical activity were collected using an Actigraph GT1M accelerometer which participants wore on a waistband for 7 days immediately after enrolment and for 7 days immediately before the follow-up clinic at 6 months. Only when there were five or more valid wear days (when the accelerometer detected it was being worn for at least 10 h) were the data used in analysis. Accelerometer non-wear time was defined as a run of 60 min of consecutive zero counts, consistent with other studies on older adults [20], allowing for a 2-min interruption, and the spurious value detection was set at 20,000 counts, based on 60-s epochs. Sociodemographic data were collected on housing tenure, marital status, and social class (including years of full-time, continuous education), in addition to smoking status and alcohol intake as lifestyle variables. Variables likely to be secondary outcomes of interest in a definitive trial were explored at baseline and 6-month follow-up and included grip strength measured using the Jamar Hydraulic Hand Dynamometer and NIH measurement protocol [21], depression measured as a continuous variable by summing scores on the 15-point Geriatric Depression Scale (GDS) [22], self-reported physical activity using the Physical Activity Scale for the Elderly (PASE) questionnaire [23], cognitive performance using the Montreal Cognitive Assessment (MoCA) [24], BMI, self-reported disability using Lawton’s Instrumental Activities of Daily Living (IADL) [25], social support using items from the National Health and Nutrition Examination Survey, and self-reported number of daily trips made outside of the home, collected using a journey log in conjunction with the accelerometer. For the feasibility assessment of an economic evaluation, quality of life was measured using EQ5D and ICECAP-O [26–28]. Data on healthcare use were obtained by reviewing GP records for all GP face-to-face consultations, out-patient appointments, hospital admissions, and emergency appointments in the 12 months prior to the start of the study. Follow-up data on healthcare use were obtained by reviewing GP records for the same events in the period following the follow-up clinic appointment. At the end of the follow-up clinic, participants were given a gift voucher worth £15 as a token of gratitude for their contribution to the study.

### Statistical analyses

Hypothesis testing for differences between two groups was undertaken using unpaired *t* tests to compare numerical means of continuous data, and chi-squared tests for categorical data, unless the expected value in a cell was less than five, in which case Fisher’s exact test was performed. Analysis of multi-group data was conducted using chi-squared tests for categorical data and one-way analysis of variance (ANOVA) for continuous data to test for heterogeneity. The distributions of all variables were inspected using histograms. Skewed variables (e.g. age, index of multiple deprivation scores, GDS scores, and SPPB scores) were transformed using natural logarithm before hypothesis testing. Negatively skewed variables were transformed after the scales had been reversed. PASE data were analysed in three different ways: complete case analysis of unambiguous data, a “high activity” sensitivity analysis, and a “low activity” sensitivity analysis (see details below under “Missing data”). The “high activity” variable was shown to be positively skewed and was thus transformed on the natural logarithmic scale for analysis. For GDS scores, there were a substantial number of zeros, so these values were transformed to natural log (GDS score + 1). Simple descriptive statistics were used to describe the distribution and baseline variability of all outcomes of interest for the definitive trial. Analyses were conducted in Stata 14 (StataCorp LP, College Station, TX, USA); accelerometer data were processed in KineSoft 3.3.75 (KineSoft, New Brunswick, Canada).

All comparisons were analysed on an “intention to treat” basis, i.e. the random allocations were preserved, and individuals were analysed in the group to which they were originally randomised.

### Missing data

Missing data from enrolled participants at baseline were obtained, where practicable, after the clinic by telephone call. Due to the considerable volume of missing data associated with the PASE instrument, sensitivity analyses were conducted assuming two scenarios: a “high activity” scenario where all missing data were assumed to relate to the highest activity level possible on the questionnaire and a “low activity” scenario where missing data were assumed to relate to the lowest activity level. Multiple imputation was not deemed appropriate given this was a feasibility study with a small sample. Whilst multiple imputation could have been undertaken for missing data, we felt this was not appropriate given that this was a small feasibility study and the PASE questionnaire was not a primary outcome.

## Results

Data have been combined for all practices used as recruitment sites. Heterogeneity between practices was not formally assessed, but visual inspection of variables such as gender and age suggested broad homogeneity.

### Recruitment

#### Postal invitations

**Fig. 1:**
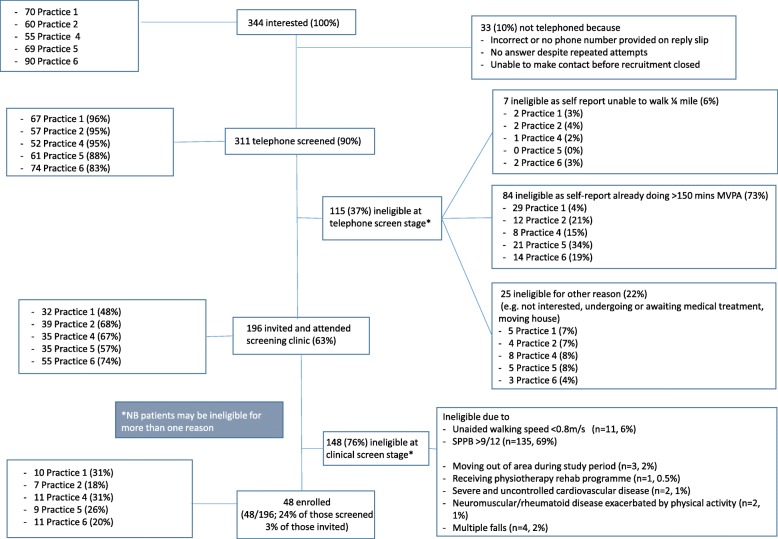
Flowchart summarising outcomes of recruitment screening for those invited by post

Of the 1884 patients identified from GP practice lists and sent an invitation in the post, responses were received from 1290 (68%). Sending a reminder increased the response rate by 25%; there was no evidence that those responding to the reminder differed in age (*p* = 0.83), gender (*p* = 0.90), or level of deprivation (*p* = 0.93) compared to those responding to the initial invitation. Only 344/1290 (27%) of the responses received were from patients interested in finding out more about the study. The major reason expressed by the 73% of respondents who were not interested in the study was that they were already meeting recommended physical activity levels (55% of those not interested in the study). Of the 344 patients interested in the study, 311 (90%) were screened by telephone with over a third (115/311, 37%) of patients found to be ineligible at this stage, mainly because they were too active (see Fig. 1). The remaining 196/311 (63%) patients were invited and attended a clinical screening appointment at their own GP practice. The numbers and proportions of patients invited by post proceeding through the recruitment and screening processes are summarised in Fig. 1.

#### Waiting room recruitment

Over the 6 days spent recruiting patients directly from the practice waiting room, 37 patients were approached, and 73% of these were not interested or reported their own ineligibility for the study. Five patients chose to take away an information pack, and none of these patients returned a response slip; five were happy to provide their contact details at the time. A further patient expressed an interest in participating after reading an information leaflet at the practice. Of these six patients, four were happy to attend a screening clinic, and of these, three (75%) were eligible and enrolled.

### Enrolment

In total, 51 participants were eligible at the clinical screening stage and all provided consent to be enrolled: 34 to the intervention arm and 17 to the control arm. There was very strong evidence that those eligible and enrolled in the study were older (74.1 v. 69.0 years) and unsurprisingly had lower scores on the SPPB and a slower walking speed compared to those not enrolled. Of the 51 participants enrolled in the pilot trial, approximately 75% were married, and there was a high level of home ownership (~ 80%) in both intervention and control arms. Six percent of the enrolled participants were non-drinkers, and this was equally balanced between intervention and control arms. Around a third of participants felt that they could have benefitted from more emotional support over the last 12 months; a minority (6%) felt that they had not had anyone to rely on for emotional support over the past 12 months. Data shown in Table 3 highlight the low volumes of moderate-to-vigorous physical activity (less than 5 min on average per day) and high levels of sedentary behaviour (8 h per day) by participants at baseline. Scores on the MoCA instrument were skewed in enrolled participants, with a median value of 25 out of 30. The proportion of participants meeting the criteria for depression was higher in the intervention arm (15%) than the control arm (6%), but there was no evidence of a difference in physical health status between participants enrolled and those ineligible for the trial.

**Table 3:** Key baseline characteristics of study population

Parameter	Measure	All enrolled	Intervention	Control
	*n* (%)	51 (100%)	34 (67%)	17 (33%)
Age at screening	Median (range)	74.1 (65.3–88.5)	73.6 (65.3–88.5)	74.8 (65.5–83.2)
Gender	No. male (%)	30 (59%)	21 (62%)	9 (53%)
IMD	Median (IQR) IMD	15.1 (4.0–33.1)	15.1 (4.6–32.6)	13.2 (6.2–23.2)
Unaided 4-m walk	Mean (SD) 4 m walking speed, unaided	0.97 (0.16)	0.96 (0.13)	1.00 (0.2)
SPPB	Median (IQR) SPPB score	9 (7–9)	9 (7–9)	9 (8–9)
GDS (depression)	Median (IQR) GDS score (depression)	2 (0–10)	2 (0–10)	2 (1–5)
PMH	Number (%) with heart disease	16 (31%)	9 (26%)	7 (41%)
	Number (%) with arthritis	21 (41%)	15 (44%)	6 (35%)
BMI	Median (IQR) BMI score	27.2 (22.3–35.5)	26.9 (22.3–34.2)	28.4 (26.0–33.1)
Marital status	Married (%)	39 (76%)	25 (74%)	14 (82%)
Smoking status	Ever smoked (%)	28 (55%)	19 (56%)	9 (53%)
Socioeconomic status	Last occupation was manual (%)	21 (41%)	15 (44%)	6 (35%)
	Owns own home (%)	42 (82%)	27 (79%)	15 (88%)
	Detached house or bungalow (%)	13 (25%)	8 (24%)	5 (29%)
MOCA	Median (IQR) MOCA score	25 (18–28)	25 (20–28)	22 (19–27)
Self-report disability	Median (IQR) Lawton’s IADL score	8 (7–8)	8 (7–8)	8 (8–8)
Social support	Can rely on someone for emotional support (%)	48 (94%)	32 (94%)	16 (94%)
	Need more emotional support (%)	14 (29%)	9 (28%)	5 (31%)
	No one to rely on to help out financially (%)	11 (22%)	7 (21%)	4 (24%)
	Median (IQR) number of close friends/family	6 (2–20)	6.5 (4–14)	6 (3–10)
	*n* (%)	50	34	16
Accelerometer-reported activity levels	Daily mean (SD) hours sedentary activity	7.9 (1.1)	7.9 (1.2)	7.9 (1.1)
	Daily mean (SD) hours in light activity	5.4 (1.2)	5.4 (1.2)	5.4 (1.2)
	Daily median (IQR) minutes in moderate-to-vigorous activity	4.5 (1–25)	6.5 (1–25)	2.5 (1–7)
	*n* (%)	48	32	16
Alcohol intake	Median (IQR) units/week in drinkers	3 (0–33)	4 (0–24)	3 (0.5–10.5)

*SD* standard deviation, *IQR* interquartile range, *IMD* index of multiple deprivation

### Adherence and retention

**Fig. 2:**
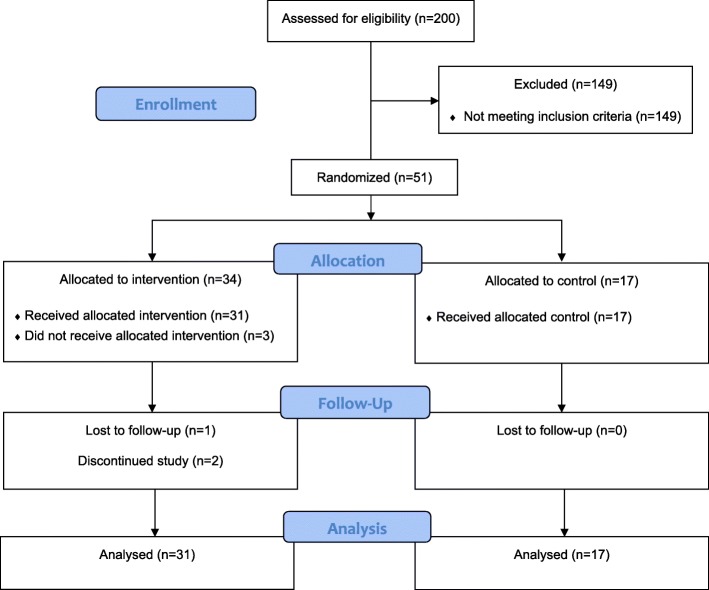
CONSORT diagram

A CONSORT diagram is presented in Fig. 2, summarising the numbers enrolled, receiving the minimum dose of intervention, and providing follow-up data. One participant died during the study, and two participants formally withdrew from aspects of the study, one less than 48 h after enrolment, citing the expected burden of paperwork, particularly the journey log as the main barrier to continued participation. During the study period, two participants from the intervention group suffered from serious health conditions which affected delivery of the intervention. Intervention sessions were temporarily suspended for both, and their participation was reinstated once the episode resolved. Overall, 43/51 (84%) of those enrolled provided a complete set of data at the 6-month follow-up clinic. A higher proportion (48/51; 92%) provided at least some follow-up data, either self-reported postal data, accelerometry, or measures at the follow-up clinic.

### Intervention dose

Most of the intervention participants (31/34, 91%) received what was deemed the “minimum dose” of sessions, at least one face-to-face and five telephone sessions, as per our a priori progression criteria (Table 1). Of the three participants not receiving the minimum dose, two had withdrawn at an early stage and one participant died during the intervention period. The mean (standard deviation) length of time between the first intervention session and the final visit was 26 (2) weeks, suggesting that the intervention sessions were delivered for the intended period of 6 months. Overall, the median amount of time spent in contact with a participant over the 6 months was 5 h, with an interquartile range from 4 to 6 h; these data were positively skewed as a minority of participants required considerably more contact time than average.

PAFs recorded the start and end times of every contact with a participant. The distributions of a number of administrative and intervention telephone calls for each participant were positively skewed, as a small number of participants required additional input and support due to the occurrence of serious adverse or life events. Administrative calls were made by PAFs on an ad hoc basis, e.g. re-arranging appointments or collecting further details of adverse events. Table 4 shows the number and duration of these contacts.

**Table 4:** Contacts with PAF

	Average number (IQR/SD)	Mean duration (SD), minutes
Administrative telephone calls	4 (1–8)	4 (2)
Telephone sessions	8 (7–11)	18 (4)
Face-to-face session	3 (0.5)	45 (8)

*IQR* interquartile range, *SD* standard deviation

### Feasibility of collecting outcome measures

Most outcome measures were collected comprehensively and accurately at the baseline and follow-up clinics; however, some survey items were associated with missing data. At baseline, only 174/200 (87%) of participants provided PASE data that was accurate or required only minor re-coding; at follow-up, this proportion was even lower at 32/47 (68%). No obvious patterns were noted in the missing PASE data; they did not appear to be related to sensitive questions. GDS scales returned by research nurses/HCAs after the follow-up appointments were observed to have more missing/double answers than those at baseline (6% v. 0%).

Fifty of the 51 participants (98%) wore the accelerometer at baseline, with only one participant finding the requirements too demanding. All accelerometers sent out to participants before their follow-up appointments were returned, with 44/48 (92%) of participants wearing one. Data retrieved from the accelerometers revealed that of the accelerometers returned at baseline 48/50 (96%), participants had worn it for at least one valid day, as detected by motion counts. At follow-up, the proportion was similar at 43/44 (91%). At baseline 42/50 (84%), participants returning a worn accelerometer had worn it for at least five valid days; at follow-up, this proportion remained high at 39/44 (89%).

Healthcare utilisation data were available for 46 participants at baseline and 39 participants at follow-up (Table 5); rates per person-year were calculated. Hospital and urgent care attendances, including A&E, out-of-hours GP services, and rapid access chest pain clinics, were rare, and the small sample size resulted in large confidence intervals.

**Table 5:** Rates of healthcare utilisation

	Baseline				Follow-up			
	Intervention (*n* = 30)		Control (*n* = 16)		Intervention (*n* = 24)		Control (*n* = 15)	
	Rate/py	95% CI	Rate/py	95% CI	Rate/py	95% CI	Rate/py	95% CI
GP appointments	5.02	4.28, 5.89	4.49	3.56, 5.65	3.48	2.13, 5.68	3.46	1.96, 6.09
Hospital admissions	1.53	1.15, 2.04	1.81	1.26, 2.60	2.17	1.17, 4.04	1.73	0.78, 3.85
Outpatient appointments	0.13	0.05, 0.35	0	na	0.22	0.031, 1.5	0	na
Urgent Care	0.70	0.46, 1.07	0.50	0.25, 1.00	0.43	0.11, 1.7	0.29	0.04, 2.1

*py* person-year, *CI* confidence interval

Additional file 2: Table S1 shows the point estimates and variability for primary and secondary outcomes, as likely to be primary outcomes in a definitive trial. One participant in the control arm suffered from a stroke and was unable to attempt the walk test at follow-up; hence, *n* = 15 for the follow-up. Additional file 2: Table S1 also contains the effect estimates (mean difference or odds ratio), confidence interval, and *p* value for each outcome, calculated in fully adjusted regression models. Whilst there appears to be moderately strong evidence against the null hypothesis for several of these outcomes, the study was not powered to detect such differences, and therefore, these should not be considered as evidence of effectiveness and the results should be treated with caution.

### Variability estimates for key outcomes

A putative sample size calculation was performed to estimate the numbers required for a definitive trial, based on an assumed primary outcome of walking speed (standard deviation of walking speed of 0.2, as shown in Additional file 2: Table S1), an alpha level of 0.05, and a beta of 0.90. The minimal clinically important difference (MCID) was obtained from work published by the LIFE study team, who used data from over 400 participants to identify the MCID for walk speed using a five-point self-reported assessment of mobility ability as anchors. An increase of between 0.03 and 0.05 m/s in 4-m walking speed was deemed to be the minimum change associated with clinically significant improvement [29]. Sample sizes were calculated for each end of the range of MCIDs (see Table 6).

**Table 6:** Sample size requirements according to different MCIDs and power levels for walking speed

	MCID on 4MWT	
MCID	0.03 m/s	0.05 m/s
Power 80%	351	128
Power 90%	469	171

*MCID* minimal clinically important difference, *m/s* metres per second

In this pilot study, 48/51 (94%) of participants who were enrolled provided some level of follow-up data, so sample size calculations should be adjusted for an assumed loss to follow-up rate of 10%, and if a definitive RCT was undertaken in primary care, it would also be necessary to consider adjusting the sample size to account for clustering by practice and household.

### Feasibility criteria

The criteria pertaining to the feasibility elements of the pilot are shown in Table 1, and Table 7 shows a summary of whether and how the criteria were met based on our findings presented in this paper.

**Table 7:** Assessment of feasibility criteria

Criteria for proceeding to a definitive trial	Assessment	Criterion fulfilled?	Evidence
An acceptable proportion of individuals respond to recruitment invitations.	Recruitment records show an initial response rate of > 10% to postal invitation and/or > 25% to primary care practitioner invitation or waiting room recruitment. The three methods will be compared.	Yes	Response to postal invitation was high at 68% overall and 27% of responders interested and self-reporting as eligible for the study.
An acceptable proportion of individuals responding to recruitment invitations are eligible to participate.	Screening records show a screen-failure rate of < 80%.	Yes	The screen-failure rate at clinical screening was 74%.
Attrition from the exploratory pilot trial is low.	Trial records show that the proportion of enrolled participants “lost to follow-up” at 6 months is < 20%, excluding deaths and long-term care or hospitalisation.	Yes	Excluding deaths and formal withdrawals, only one participant was lost to follow-up at 6 months (2%).
PAF intervention attracts high rates of participation from eligible adults.	Trial records indicate that adherence to the intervention is high; ≥ 65% of intervention participants participate in at least one face-to-face and five telephone sessions.	Yes	Adherence was very high; 91% of intervention participants received at least one face-to-face and five telephone sessions.
PAF can engage individuals from a range of socio-economic localities.	Participants are recruited from primary care practices in wards with high deprivation scores and low deprivation scores.	Yes	Practices and participants from a variety of sociodemographic backgrounds were involved in the study.
PAF delivery costs can be recorded in a way that enables cost-effectiveness analysis.	Systems developed in the exploratory trial can be used to monitor the costs of a definitive RCT.	Yes	Collection of data on PAF contact time is feasible and could be employed alongside standard trial methods for monitoring costs.
Methods for measuring primary and secondary outcomes and mediator variables are feasible and acceptable.	Process evaluation findings and completed questionnaires suggest that self-report and objective measures were comprehensible and acceptable to > 80% of participants.	Yes—with modification (PASE questionnaire)	Measurement of outcomes was acceptable and largely feasible. However, the pilot study revealed issues with some self-report instruments and ascertainment of these outcomes would need to be modified in any potential future definitive trial.
The sample size required for an adequately powered trial is achievable.	Measurement variability of the primary outcome, recruitment rates, and expected attrition are consistent with a sample size that can be achieved within a reasonable time.	Yes	Sample size calculations under a range of parameters all suggest a definitive trial is achievable.

## Discussion

The study reported in this paper was designed to assess the feasibility of running a definitive RCT of the PAF intervention, designed to improve physical function in older adults, in a primary care setting. Findings presented here reveal that our a priori progression criteria for the trial processes have been met, and therefore, it is feasible to deliver a trial of this nature. Postal recruitment was effective and feasible to deliver at scale, and the procedures for two-stage screening were successful in identifying a sample of patients meeting the eligibility criteria. Most outcome measures were collected successfully at baseline and follow-up; accelerometers were well tolerated, and this method is viable for collecting objective measurement of physical activity.

### Strengths

By asking PAFs to complete diaries, we could assess the feasibility of collecting accurate data on the cost of the intervention; the completeness of these diaries and the low rates of missing data in the EQ5D and ICECAP-O survey items used to calculate QALYs make economic evaluation a feasible component of a definitive trial. A further strength of the PAF diaries is that they enabled us to understand whether participants were receiving the correct “dose” of intervention and to identify and explore the reasons behind a very small number of participants receiving very high levels of contact (data to be published separately). Collection of data from the primary care record on healthcare utilisation was also shown to be feasible, though further work should validate this data source and explore methods for automation (e.g. using linked datasets) which would reduce time burden and user error.

Recruitment from six different GP practices and involvement of more than one PAF in delivery of the intervention are further strengths, as both highlight the generalisability of setting and agent of change.

Moreover, the pilot RCT was sufficiently large as to enable estimation of the samples size that would be required to conduct an adequately powered definitive trial using a primary outcome that we have tested and shown to be feasible.

### Lessons learned

Whilst our findings are positive, there are some useful learning points from this pilot study which should be considered when designing a definitive trial. Waiting room recruitment was not deemed to be an effective strategy in this population, despite reports by others of its effectiveness [30]. This may be due to the age of the population targeted, who are likely to have more significant health concerns than younger adults and therefore may not be as receptive to discussing research participation whilst awaiting a GP consultation.

Whilst procedures for screening were implemented with few problems overall, the screen-positive rate of 26% is low. The time taken to screen potential participants was therefore greater than anticipated, so the recruitment closed before reaching the desired 60 participants; however, as this was an unpowered pilot study, the total sample size was not critical to the study. Improved methods for selecting patients to screen would be required to make this stage more manageable and cost-effective in a definitive trial. Recruitment to this pilot trial by postal invitation involved 1884 invitations which were sent to recruit 48 participants, an overall recruitment rate of 3%. Our sample size calculation suggests that a final sample of between 128 and 469 participants would be required for a definitive trial. Employing the same recruitment approach as in this pilot study would therefore require postal invitations to be sent to between 4267 and 15,633 people and between 533 and 1954 people to be clinically screened in order to meet the sample requirement. As this is an inefficient approach to recruitment, an alternative approach will be required. Potential solutions to improve recruitment efficiency include better targeting of invitations to patients at risk of disability, e.g. through identifying read codes which are predictive of functional ability, by raising the age threshold to 70 or 75 years, and by providing clearer and more informative eligibility descriptions in the recruitment materials. Given the large proportion of responses from patients who were self-reporting ineligibility (55% of postal reply slips and 27% of those screened by telephone), increasing the specificity of self-reported eligibility would reduce the volume of responses from patients who had a high level of physical function at baseline. It has been shown that walking speed alone can represent nearly all of the predictive value of the SPPB [31], is quick and low cost, and may be done outside of a clinical setting. There has also been some work to validate self-reported performance on tests of physical performance, using a “virtual SPPB” test [32]. It would be feasible to design an app that participants downloaded and allowed them to test their walking speed with automatic notification to the study team as to whether they were eligible or not. Whether such an approach is acceptable and cost-effective in recruiting this population is unknown but testable. Simplifying and streamlining the approach to identifying the eligible population would reduce the overall workload and cost of recruitment.

Most of the data were collected successfully as nominated primary and secondary outcomes for a definitive trial; however, the PASE tool, used to measure self-reported physical activity, had high levels of missing data. Given the low correlation between self-reported and objectively measured physical activity [33], the role of self-reported physical activity in trials with older adults at all is questionable. Furthermore, self-reported physical activity measures are dependent upon high-level cognitive processes to accurately recall details and timings about activities in the past; with older adults, misclassification due to mild cognitive impairment may be increasingly likely. Careful consideration should be given to whether self-reported physical activity would add any value to a definitive trial.

## Conclusions

We conclude that it is feasible and practical to deliver an RCT of a one-to-one behavioural physical activity intervention like the PAF intervention described here, in a population of older adults at risk of disability. To improve the efficiency of the recruitment process, greater specificity in identifying the target population is required and we have suggested several approaches to doing this. It is generally agreed that tackling the burden of age-associated dependency will require a multi-level approach including effective treatment of chronic diseases, reduction in risk factors for cardio- and cerebrovascular disease (e.g. hypertension, hypercholesterolemia, tobacco use), efficient care packages, reduced reliance on care homes, and encouraging and supporting older adults to remain economically active for longer [34], where appropriate. Nevertheless, this pilot trial shows that it would be feasible to evaluate one approach to risk factor reduction, and if an intervention such as PAF can show similar effects to the LIFE study, but at lower cost, then this has the potential to have a considerable impact at a population level.

## Additional files

Additional file 1: **Figure S1.** Screening flowchart. (PNG 78 kb)

Additional file 2: **Table S1.** Complete data on estimates and regression outputs for primary and secondary outcomes. (DOCX 27 kb)
