# Supplementary figures and images for: A pilot randomised controlled trial of physical activity facilitation for older adults: feasibility study findings
Source: Pilot Feasibility Stud. 2019 Mar 8;5:40. doi: 10.1186/s40814-019-0414-9 (PMC6407174; doi:10.1186/s40814-019-0414-9)

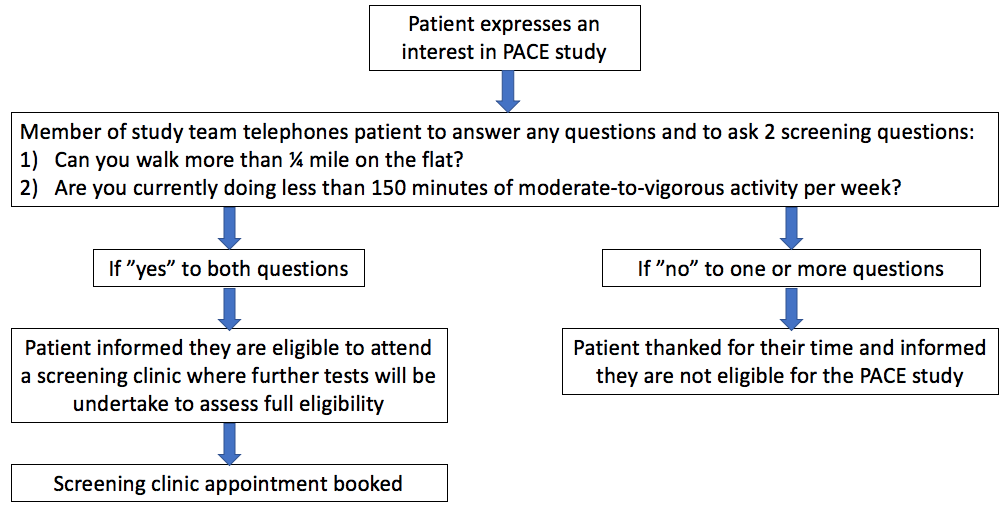

Supplement: Supplementary file 1 — Figure S1. Screening flowchart. (PNG 78 kb) [file 40814_2019_414_MOESM1_ESM.png]
