# Supplementary material for: A pilot randomised controlled trial of physical activity facilitation for older adults: feasibility study findings
Source: Pilot Feasibility Stud. 2019 Mar 8;5:40. doi: 10.1186/s40814-019-0414-9 (PMC6407174; doi:10.1186/s40814-019-0414-9)
Supplement: Supplementary file 2 — Table S1. Complete data on estimates and regression outputs for primary and secondary outcomes. (DOCX 27 kb) [file 40814_2019_414_MOESM2_ESM.docx]

Table S1 Complete data on estimates and regression outputs for primary and secondary outcomes

|  | **Baseline** | | **Follow-up** | | **EE** | **95% CI** | **p** |
| --- | --- | --- | --- | --- | --- | --- | --- |
|  | **I** | **C** | **I** | **C** |  |  |  |
| **n** | **34** | **17** | **31** | **15** | **Mean difference** | | |
| mean (sd) 4m walking speed, unaided | 0.96 (0.1) | 1.0 (0.2) | 1.12 (0.2) | 1.03 (0.2) | 0.13 | -0.00-0.26 | 0.06 |
| median (IQR) SPPB score | 9 (7-9) | 9 (8-9) | 9 (7-10) | 10 (9-11) | -0.54 | -1.42-0.34 | 0.22 |
| **n** | **34** | **17** | **31** | **16** | **Odds ratio** | | |
| SPPB < 9/12 | 34 | 17 | 16 | 6 | 2.35 | 0.45-12.2 | 0.31 |
| mean (sd) grip strength | 25.2 (10.8) | 22.3 (8.6) | 25.6 (10.6) | 23.3 (10.1) | -0.38 | -3.92-3.17 | 0.83 |
| median (IQR) MOCA score | 25 (20-28) | 22 (19-27) | 27 (20-30) | 24.5 (20-28) | -0.07 | -2.06-1.91 | 0.94 |
| median (IQR) BMI | 26.9 (22-34) | 28.4 (26-33) | 26.3 (23-35) | 27.6 (25-34) | 0.09 | -0.88-1.06 | 0.85 |
| median (IQR) Lawton's IADL score | 8 (7-8) | 8 (8-8) | 8 (7-8) | 8 (7-8) | 0.48 | -0.19-1.15 | 0.16 |
| median (IQR) no. daily trips | 1.5 (0.9-2.8) | 1.4 (0.8-2.4) | 1.4 (0.8-3) | 1.4 (0.7-1.7) | 1.16 | 0.82-1.64 | 0.38 |
| **n** | **28** | **14** | **27** | **12** | **Mean difference** | | |
| Median daily mins MVPA | 6.5 (1-25) | 2.5 (1-7) | 4 (1-37) | 8 (5-11) | -0.92 | -8.81-6.98 | 0.81 |
| Mean daily mins light physical activity | 324.4 (74.0) | 325.3 (74.5) | 367.4 (83.6) | 335.2 (79.7) | -7.55 | -44.93-0.55 | 0.68 |
| Median daily minutes of sedentary activity | 495.5 (396 - 555) | 505.5 (432-526) | 496 (407-548) | 492 (444-498) | 9.37 | -23.2-41.9 | 0.56 |
| **n** | **31** | **15** | **19** | **13** | **Mean difference** | | |
| mean (sd) self-report PA level (PASE) score | 112.9 (52.5) | 112.1 (50.0) | 112.4 (52.4) | 133.5 (76.9) | -55.6 | -108.0--3.2 | 0.04 |
| **n** | **34** | **17** | **30** | **17** | **Mean difference** | | |
| median PASE score: for missing data assume high PA | 118.5 (51-197) | 110 (96-162) | 131.5 (47-231) | 167 (89-215) | -23.3 | -84.9-38.4 | 0.45 |
| mean PASE score: for missing data assume low PA | 116.0 (52.5) | 115.9 (46.9) | 112.9 (52.9) | 133.7 (69.0) | -36.7 | -69.9--3.58 | 0.03 |
| **n** | **34** | **17** | **28** | **16** | **Mean difference** | | |
| median (IQR) GDS score | 2 (0-10) | 2 (1-5) | 2 (0-9) | 2 (1-6) | -0.5 | -1.63-0.63 | 0.38 |

I: intervention C: control IQR: inter-quartile range PA: physical activity

sd: standard deviation EE: effect estimate CI: confidence interval
